# Demographic and socioeconomic inequalities in the risk of emergency hospital admission for violence: cross-sectional analysis of a national database in Wales

**DOI:** 10.1136/bmjopen-2016-011169

**Published:** 2016-08-24

**Authors:** Sara Jayne Long, David Fone, Andrea Gartner, Mark A Bellis

**Affiliations:** 1 DECIPHer, UKCRC Centre of Excellence, Cardiff University, Cardiff, UK; 2 Division of Population Medicine, Cardiff University, Cardiff, UK; 3 Public Health Wales, Cardiff, UK

**Keywords:** Violence, Socio-economic risk, Injury, Emergency admission

## Abstract

**Objectives:**

To investigate the risk of emergency hospital admissions for violence (EHAV) associated with demographic and socioeconomic factors in Wales between 2007/2008 and 2013/2014, and to describe the site of injury causing admission.

**Design:**

Database analysis of 7 years’ hospital admissions using the Patient Episode Database for Wales (PEDW).

**Setting and participants:**

Wales, UK, successive annual populations ∼2.8 million aged 0–74 years.

**Primary outcome:**

The first emergency admission for violence in each year of the study, defined by the International Classification of Diseases V.10 (ICD-10) codes for assaults (X85-X99, Y00-Y09) in any coding position.

**Results:**

A total of 11 033 admissions for assault. The majority of admissions resulted from head injuries. The overall crude admission rate declined over the study period, from 69.9 per 100 000 to 43.2 per 100 000, with the largest decrease in the most deprived quintile of deprivation. A generalised linear count model with a negative binomial log link, adjusted for year, age group, gender, deprivation quintile and settlement type, showed the relative risk was highest in age group 18–19 years (RR=6.75, 95% CI 5.88 to 7.75) compared with the reference category aged 10–14 years. The risk decreased with age after 25 years. Risk of admission was substantially higher in males (RR=4.55, 95% CI 4.31 to 4.81), for residents of the most deprived areas of Wales (RR=3.60, 95% CI 3.32 to 3.90) compared with the least deprived, and higher in cities (RR=1.37, 95% CI 1.27 to 1.49) and towns (RR=1.32, 95% CI 1.21 to 1.45) compared with villages.

**Conclusions:**

Despite identifying a narrowing in the gap between prevalence of violence in richer and poorer communities, violence remains strongly associated with young men living in areas of socioeconomic deprivation. There is potential for a greater reduction, given that violence is mostly preventable. Recommendations for reducing inequalities in the risk of admission for violence are discussed.

**Strengths and limitations of this study**

- This study used a total population-based approach, over a 7-year time period, and included a large enough number of emergency admissions to facilitate a fully stratified descriptive analysis and a robust modelling approach.
- Hospital admission data are important for measuring the impact of violence on health and for assessing the effectiveness of interventions in policy and practice, but such data alone are not sufficient. Many assaults are dealt with by ambulance services, emergency departments, general practitioners, or by the individual or their family. A more comprehensive understanding of violence can be generated by using a multiagency approach.
- This cross-sectional design does not identify cause and effect.

## Introduction

Violence is a major cause of morbidity and mortality worldwide. Using data from 133 countries, the WHO^1^ estimated that violence, with all categories combined (self-directed, interpersonal and collective), accounts for 2.5% of global mortality, or 1.3 million deaths annually. The focus of this study is on interpersonal violence, that is, violence among family members, intimate partners, friends, acquaintances and strangers. However, the terms violence and interpersonal violence are used interchangeably throughout. Death from violence occurs in only a minority of cases; violence is linked to a range of non-fatal physical injuries with injuries to the head and neck the most common injury type.^2^ In addition, violence is associated with negative social and mental health outcomes.^1^ ^3^ The health consequences of violence summarised by the WHO^1^ are outlined in box 1.

Box 1 **Behavioural and health consequences of violence**

*Physical*: abdominal injuries, thoracic injuries, brain injuries, burns/scalds, fractures, lacerations, disability;

*Mental health and behavioural*: alcohol and drug abuse, depression and anxiety, post-traumatic stress disorder, eating and sleeping disorders, attention deficits, hyperactivity, externalising behaviour, smoking, suicidal thoughts, suicidal behaviour, unsafe sex;

*Sexual and reproductive health*: sexually transmitted infections, unintended pregnancy, pregnancy complications, chronic pelvic disease;

*Chronic disease*: arthritis and asthma, cancer, cardiovascular disorders, diabetes, kidney problems, liver disease, stroke.

Such outcomes present by far the greatest burden to health and social care systems.^1^ ^3–5^ Thus, the economic costs of violence are substantial. Direct costs include the provision of treatment, mental health services, emergency care and criminal justice responses, but there are also a range of indirect costs. Victims are more likely to experience periods of unemployment, absenteeism and to suffer health problems that affect job performance.^1^ Other indirect costs include long-term disability, social care costs attributable to safe accommodation for women and children, dysfunctional lifestyle due to fears for personal safety and a lack of investment in areas with the highest rates of violence.^1^ An independent analysis by the London School of Economics estimated the total economic and social costs of violence in 2008/2009, including domestic violence, to be £29.9 billion.^5^ ^6^ Most of these costs fell on the victims of crime, but there were also costs to the public sector. For example, the cost to the National Health Service (NHS) of providing services related to the physical and mental health consequences of violent crime was estimated at around £2.9 billion. The costs to the criminal justice system were £4.3 billion.

Interpersonal violence has one of the strongest inequality gradients.^7–13^ Most deaths from violence occur in the poorest countries; however, non-fatal outcomes present a substantial burden even in high-income countries where experiences of violence are strongly related to deprivation. In a 5-year ecological study, Bellis *et al*^7^ examined relationships between emergency hospital admissions for violence (EHAV), deprivation, age (0–74 years) and gender in England. The odds of admission for violence increased with increasing quintiles of deprivation and were 5.5 times higher in the most deprived quintile compared with the least. In males aged 17–19 years, violence accounted for 20% of the difference between the most and least deprived quintiles in all-cause emergency hospital admissions.

Another English study found significant relationships between deprivation and parental investigation for child maltreatment and for registration of children (aged under 6) with child protection services.^8^ Children exposed directly to, or as witnesses to recurrent violence are also more likely to develop both antisocial and health harming tendencies themselves across their life course.^8^

Individuals living in deprived areas (particularly young men) have significantly higher risks of hospitalisation for violent injury (England)^10^ and violent death (Scotland).^11^ In Canada, persons requiring emergency medical care for injuries resulting from violence resided most commonly in areas of social deprivation.^12^ International reviews highlight consistent association between deprivation and risks of being both a perpetrator and a victim of violence,^13^ ^14^ and the authors expressed a need to address inequalities at national and international levels.

Although a growing number of studies demonstrate that violence is preventable, investment in violence prevention is not on a scale that matches the economic burden to health and social care.^5^ Large-scale evidence reviews from the WHO have identified 7 ‘best buy’ strategies,^1^ 6 focusing on prevention and 1 focusing on immediate response (box 2). The prevention and reduction of violence features in 17 of the 169 targets in United Nations (UN) Sustainable Development Goals (SDGs).^15^ ^16^

Box 2 **WHO strategies to prevent and reduce violence**

1. Developing safe, stable and nurturing relationships between children and their parents and caregivers;

2. Developing life skills in children and adolescents;

3. Reducing the availability and harmful use of alcohol;

4. Reducing access to guns and knives;

5. Promoting gender equality to prevent violence against women;

6. Changing cultural and social norms that support violence;

7. Victim identification, care and support programmes.

A more detailed exploration of the differences in the prevalence of violence by sociodemographic factors (age, gender, deprivation quintile and rural–urban status) is required to inform more targeted intervention and prevention activity. The aim of this study is to investigate the risk of EHAV associated with demographic and socioeconomic factors in Wales between 2007/2008 and 2013/2014, and to describe the site of injury resulting in admission.

## Methods

### Design and study population

Emergency hospital admissions data for violence were extracted from the Patient Episode Database for Wales (PEDW). This is a population-based database of all episodes of inpatient and day case activity in NHS Wales hospitals, and for Welsh residents treated in other UK nations (primarily England). PEDW includes both planned and emergency admissions, and each consultant episode contains demographic details, clinical coding of diagnoses and operative procedures, and the postcode of residence that can be geocoded into 1 of the 1909 lower layer super output areas (LSOAs) based on the 2011 UK Census. LSOAs are small geographical areas with a mean population of 1500 people that were designed to improve the reporting of small area statistics in England and Wales. PEDW records were extracted for the financial years (from April to March) 2007/2008 to 2013/2014 if the admitting episode contained the International Classification of Diseases V.10 (ICD-10) codes for assaults in any coding position (X85-X99, Y00-Y09; ie, all types of assaults including by bodily force, sharp object, blunt object, firearm, chemical or other means). Patients were counted once per financial year, and where there was more than one admission then we selected the first in the financial year. Persons were included if they were aged 0–74 years and resident in Wales at the time of admission. Persons aged 75 years and over were excluded due to small numbers of events. Non-Wales residents attending a hospital in Wales were also excluded from the analysis.

### Statistical analyses

Counts of admissions were stratified by age, gender, LSOA deprivation quintile (assigned using the Welsh Index of Multiple Deprivation (WIMD, 2014),^17^ and the Office for National Statistics (ONS) settlement type (urban (population over 10 000); rural town and fringe; rural village and dispersed).^18^ Mid-year population estimates from ONS^18^ stratified by age group, gender, deprivation quintile and settlement type were used to calculate rates. Age categories were created based on 5-year ONS age bands, starting with those under 1 year of age and further subdividing the 15–19 age group to distinguish children aged 15–17 years from adults aged 18 years and over. Site of injury codes were extracted and summarised (ICD-10 S00-T14).

To explore the pattern of emergency admissions for violence across age groups and deprivation quintiles between 2007/2008 and 2013/2014, crude rates (per 100 000) of emergency admissions were calculated for each year, separately for males and females. The change in admissions over the study period for males and females in each deprivation quintile was calculated by subtracting 2013/2014 from 2007/2008, and the change attributable to each quintile was calculated as a proportion of the overall change. Relative risks (RRs) with 95% CIs were estimated using a generalised linear model of counts of admissions with a negative binomial log link, with age group, gender, deprivation quintile and settlement type entered as categorical covariates and a population offset. We also fitted a term for year to allow for an annual trend. Participants who were admitted in more than 1 year were included in the analysis for each year that they attended to facilitate an annual comparison of admission rates. Since a participant is likely to have a higher risk of a second admission given that they have had a first, and that this risk might vary by socioeconomic factors, we assessed the impact of this possible bias by repeating the analysis with each individual participant included only once over the study period. All analyses were performed using SPSS V.22 (IBM SPSS Statistics (http://www-01.ibm.com/software/analytics/spss/products/statistics/)).

## Results

**Figure 1:**
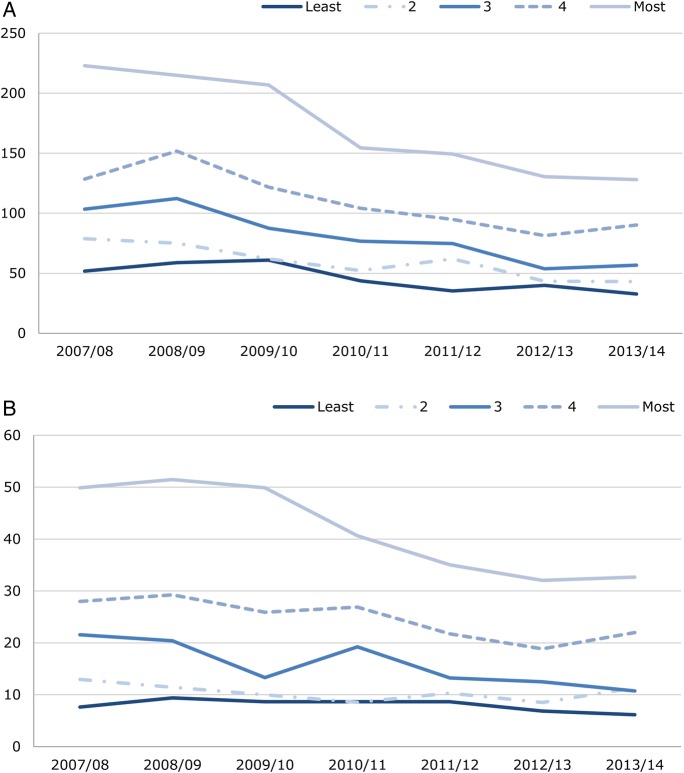
(A) Males and (B) females with an emergency hospital admission for assault, crude rate per 100 000 by deprivation fifth, financial years 2007/2008–2013/2014. Produced by Public Health Wales using PEDW and MYE (ONS), WIMD 2014 (WG)*.

**Figure 2:**
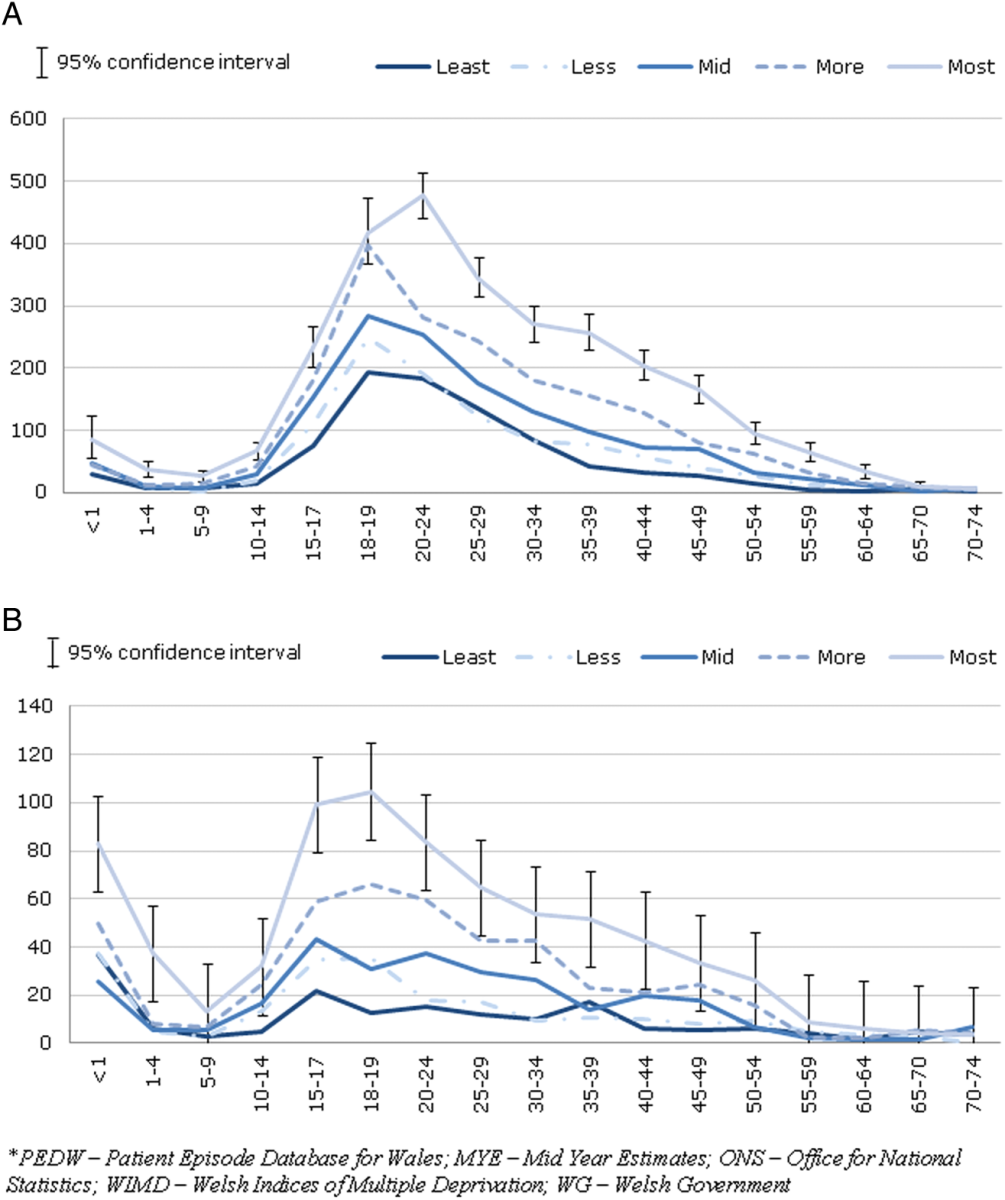
(A) Males and (B) females with an emergency hospital admission for assault, crude rate per 100 000 by deprivation quintile and age, Wales, financial years 2007/2008–2013/2014. PEDW and MYE (ONS), WIMD 2014 (WG)*.

A total of 11 033 persons aged under 75 years were admitted with an assault code. Of these, 389 (3.5%) were admitted in more than 1 year. The overall admission rate declined over the study period, from 69.9 per 100 000 population to 43.2 per 100 000 (table 1). This decline was observed in males (116.3–70.0) and females (23.9–16.5). In males, over one-third (−39.2%) of the change in admissions over the study period was attributable to a decrease in admissions in the most deprived quintile (−15.8%, −20.9%, −15.7% and −8.3% for decreasing quintiles of deprivation, respectively; figure 1). For females, almost one-half (−45.9%) of the change was attributable to a decrease in the most deprived quintile (−15.3%, −30.6%, −4.1% and −4.1% for decreasing quintiles of deprivation, respectively; figure 1). Hence, the greatest decline in admissions for violence was observed in the most deprived quintile. Among males, the peak between the ages of 18 and 24 years was substantially larger in the most deprived compared with the least deprived quintile (figure 2), suggesting that this age group accounts for the greatest inequality. The pattern was slightly different for females, where admissions peaked between the ages of 15 and 19 years (figure 2).

**Table 1:** Risks of emergency admission for violence by demographic and socioeconomic factors

Characteristic	Admitted for violence (N)	Population	Crude rate per 100 000 population	Adjusted relative risk* (95% CI)
Year				
2007/2008	1925	2 753 927	69.9	REF
2008/2009	2021	2 771 281	72.9	1.04 (0.96 to 1.12)
2009/2010	1784	2 782 161	64.1	0.89 (0.82 to 0.97)
2010/2011	1485	2 789 768	53.2	0.77 (0.71 to 0.84)
2011/2012	1409	2 800 068	50.3	0.73 (0.67 to 0.80)
2012/2013	1194	2 806 329	42.5	0.61 (0.56 to 0.66)
2013/2014	1215	2 811 275	43.2	0.62 (0.57 to 0.68)
Age				
0	126	246 326	51.2	1.78 (1.44 to 2.21)
1–4	148	972 684	15.2	0.53 (0.43 to 0.65)
5–9	111	1 162 534	9.5	0.34 (0.27 to 0.42)
10–14	344	1 251 774	27.5	REF
15–17	835	810 580	103.0	3.83 (3.32 to 4.41)
18–19	1044	580 764	179.8	6.75 (5.88 to 7.75)
20–24	2377	1 459 297	162.9	5.98 (5.25 to 6.81)
25–29	1612	1 290 472	124.9	4.35 (3.81 to 4.97)
30–34	1108	1 206 871	91.8	3.27 (2.85 to 3.76)
35–39	968	1 316 257	73.5	2.64 (2.30 to 3.04)
40–44	858	1 496 691	57.3	2.12 (1.84 to 2.44)
45–49	686	1 512 619	45.4	1.72 (1.49 to 1.99)
50–54	391	1 391 038	28.1	1.10 (0.93 to 1.28)
55–59	201	1 337 015	15.0	0. 58 (0.48 to 0.69)
60–64	120	1 380 296	8.7	0.35 (0.28 to 0.43)
65–69	63	1 163 422	5.4	0.22 (0.17 to 0.29)
70–74	41	936 169	4.4	0.18 (0.13 to 0.25)
Gender				
Female	1961	9 785 782	20.0	REF
Male	9072	9 729 027	93.2	4.55 (4.31 to 4.81)
Deprivation quintile				
Least deprivation	1039	3 858 746	26.9	REF
Less deprivation	1373	3 928 533	34.9	1.32 (1.21 to 1.45)
Mid deprivation	1932	4 003 610	48.3	1.75 (1.61 to 1.90)
High deprivation	2632	3 904 277	67.4	2.35 (2.16 to 2.55)
Highest deprivation	4057	3 819 643	106.2	3.60 (3.32 to 3.90)
Rural/urban settlement type				
Urban	8555	13 308 802	64.3	1.37 (1.27 to 1.49)
Rural town and fringe	1678	3 362 206	49.9	1.32 (1.21 to 1.45)
Rural village and dispersed	800	2 843 801	28.1	REF

*Adjusted for year, age, sex, deprivation and rural/urban.

The majority of admissions for assault resulted from head injuries (table 2). In males, the commonest injuries were fractures of the skull and facial bones (n=2962) of which 1745 were fractured mandibles (lower jaw), a further 604 were fractures of the zygomatic (cheek bone) and maxillary bones (upper jaw) and there were 113 skull fractures. Superficial and open wounds accounted for 854 of head injury admissions. The proportion of head injuries was smaller in females, with a smaller proportion of fractures and a higher proportion of superficial and open wounds. Males were more likely to be admitted with injuries to the wrist and hand, and females were more likely to be admitted for injuries to the abdomen, lower back, lumbar spine and pelvis, the elbows and forearm. Almost one-third of injury sites were grouped as ‘other’ in females compared with 1 in 15 grouped as ‘other’ among males.

**Table 2:** Injury site in emergency admissions for assault by primary diagnosis, financial years 2007/2008–2013/2014

		Males		Females	
	ICD-10 codes	Number	Per cent	Number	Per cent
Injuries to the head	S00-S09	6389	70.4	863	44.0
Injuries to the neck	S10-S19	98	1.1	23	1.2
Injuries to the thorax	S20-S29	312	3.4	59	3.0
Injuries to the abdomen, lower back, lumbar spine and pelvis	S30-S39	290	3.2	126	6.4
Injuries to the shoulder and upper arm	S40-S49	167	1.8	44	2.2
Injuries to the elbow and forearm	S50-S59	219	2.4	68	3.5
Injuries to the wrist and hand	S60-S69	553	6.1	84	4.3
Injuries to the hip and thigh	S70-S79	70	0.8	25	1.3
Injuries to the knee and lower leg	S80-S89	279	3.1	40	2.0
Injuries to unspecified part of trunk, limb or body region	S90-S99	14	0.2	12	0.6
Injuries involving multiple body regions	T00-T07	28	0.3	12	0.6
Injuries to unspecified part of trunk, limb or body region	T08-T14	26	0.3	12	0.6
All other		627	6.9	593	30.2
Total		9072	100	1961	100

ICD-10, International Classification of Diseases V.10.

The fully adjusted model showed a significant declining trend in the risk of admission over the study period (table 1). Compared with the reference age group category of 10–14 years, the RR of admission was lower in children aged 1–9; however, infants aged under 1 year had a significantly greater risk of being admitted (table 1). The risk increased substantially from age 15 years, and peaked in the age group 18–19 years when it was over six times higher. There was a decline in the risk of being admitted for violence over the age of 25 years, although the risk was still significantly higher until the age of 55 years after which the risk was significantly lower. The risk of admission was substantially higher in males, for residents of the most deprived areas of Wales (despite a decline in admissions in the most deprived quintile over the study period, overall, the risk of admission was 3.6 times greater than the least deprived quintile), and higher in cities and towns compared with living in villages. We found very little difference between these model estimates and the analysis that included only the first admission in the study period (results not shown).

## Discussion

### Principal findings

This study of the Welsh population found that the risk of EHAV was substantially higher in males and in young people aged 18–24 years. We found a substantially higher risk of admission in males and females within this age group living in areas of higher socioeconomic deprivation. Most importantly, over the study period, we found evidence of a narrowing of the existing inequality in admission rates in Wales by socioeconomic deprivation, a positive finding given that reducing inequalities is fundamental to public health practice. Despite a narrowing of inequalities, there remains a clear gradient of increasing risk associated with living in deprived areas of Wales. The risk of admission was over three times higher in the most deprived compared with the least deprived areas of Wales, and the risk was also higher in urban and town settlements than in rural villages.

The most common cause of admission was head injuries, accounting for the majority of male admissions and towards one-half of female admissions. Among males, the commonest injuries were fractures of the skull and facial bones, with the most common injury being a fractured jaw. The proportion of head injuries was smaller in females, with a higher proportion of superficial and open wounds. Males were more likely to be admitted with injuries to the wrist and hand, and females were more likely than males to be admitted for injuries to the abdomen, lower back, lumbar spine and pelvis.

### Strengths and limitations

The main strength of our study is the total population-based approach over a 7-year time period. This resulted in a large enough number of emergency admissions to facilitate a fully stratified descriptive analysis and a robust modelling approach with sufficient precision. The PEDW data set undergoes rigorous quality checking and has been used in previous epidemiological studies.^19^ ^20^ Missing data were unlikely to substantially influence the results as age and gender coding in PEDW approaches 100%, and around 0.5% of records are missing an LSOA code.^21^ A limitation of using administrative hospital admission data is that we cannot be certain about the completeness, accuracy and reliability of the clinical coding fields, given there is variation in clinical coding between hospitals. The PEDW data set does not include information on individual socioeconomic position. We were therefore unable to investigate the relationship between this, the ecological measure of deprivation assigned by residence, and the study outcomes. There is variability between studies in the extent to which individual and contextual characteristics contribute to injuries.^22^ One study reported that area socioeconomic deprivation is not a strong independent predictor of violence-related injuries in comparison to individual characteristics,^22^ whereas another study examining factors associated with injury types among adolescents revealed independent contributions of individual-level and area-level measures on hospitalised and fighting injury.^23^ A Canadian study found that among adults under 35 years, area-level socioeconomic status (SES) was a more statistically significant predictor of increased odds of assault injury than individual SES.^24^

Since the data extract included only the first admission in each year, we could not account for possible multiple admissions within each year. It is therefore likely that the admissions for assault were slightly underestimated, although we know that only around 3.5% of participants were admitted in more than 1 year. However, we found that multiple admissions in different years did not bias the risk estimates for admission as we found no discernible difference in the results between our main model and the model with each individual participant included only once over the study period.

To the best of our knowledge, this is the first UK study to quantify the site of injury among males and females resulting in an emergency admission for violence. However, nearly one-third of injuries among females were coded as ‘all other’, precluding a more detailed assessment of site of injury.

Hospital admission data are important for measuring the impact of violence on health and for assessing the effectiveness of interventions in policy and practice, but such data alone are not sufficient. Although admissions data provide an understanding of the most damaging violent events, there are many more assaults that are dealt with by ambulance services, emergency departments (EDs), general practitioners, or by the individual or their family. To generate a more comprehensive understanding of violence, a multiagency approach is required, incorporating data from the police, EDs, ambulance services and nationally collected data such as the Crime Survey for England and Wales.

### Previous research and policy

In line with previous studies, this study has shown that deprivation is strongly associated with the risk of violence,^7–14^ ^25^ including child maltreatment,^8^ knife crime^14^ and death.^11^ Two studies have examined the risk of EHAV in England.^7^ ^10^ We found similar risks of admission associated with age group, gender and socioeconomic deprivation and a decline in rates of admissions after 25 years of age. Taking the present study and the previous studies together, these data suggest that the relationship between violence and deprivation is particularly strong between the ages of 15–24 years. During young to middle adulthood (21–44 years), although there were falls in rates for violence across all levels of deprivation and in both sexes, rates were still substantially higher in the most deprived quintile. To the best of our knowledge, we have shown for the first time that this strong association persists, despite a narrowing of socioeconomic inequality over a 7-year period. It is not known if the reduction in inequality observed in the present study applies elsewhere; nevertheless there are a range of national and international policies, strategies and initiatives that may have contributed to the reduction.

Although a comprehensive review is beyond the scope of this study, an overview of key measures is considered that were implemented prior to, or during the study period (2007–2014). Interpersonal violence is strongly associated with social factors such as unemployment, income inequality, rapid social change and access to education;^26^ thus, measures that aim to reduce inequality and address such factors may indirectly impact on levels of violence. Communities First,^27^ a Welsh Government programme introduced throughout Wales in 2001, increased investment in the most deprived areas with the aim to tackle poverty by narrowing the economic, education and skills, and health gaps between the most and least deprived communities. The Flying Start programme,^28^ launched across all local authorities in Wales in 2007/2008, is a Welsh Government programme that aims to improve life chances for children from birth to age 4 by providing early years care and education. The programme delivers targeted investment in the most disadvantaged communities in Wales. Families in deprived communities are offered an enhanced health visiting service; free, high-quality, part-time childcare; and evidence-based parenting support programmes. The Welsh Governments strategic framework for Our Healthy Future^29^ was introduced in 2009 and sets the foundation for the Government's ambitions for public health, with the aim of improving the health of the Wales population.

The Welsh Government action plan, Fairer Health Outcomes For All: Moving the Agenda Forward,^30^ was introduced in 2011 and aims to tackle avoidable and unfair differences in health. It is possible that through targeted reduction in social inequalities, these strategies and initiatives had a positive impact on familial and community outcomes and indirectly influenced levels of violence.

The ‘Cardiff Model for Violence Prevention’^31^ advocates the sharing of information among emergency services, an approach found to be effective for reducing violent assault. The College of Emergency Medicine produced guidance based on the Cardiff Model, advocating the sharing of non-personal data with the police, particularly core information on the date, location and type of assault. In Cardiff, this resulted in a 35% reduction in the number of assault presentations to EDs between 2000 and 2005, compared with an 18% decrease overall in England and Wales over the same period. It is plausible that the Cardiff Model continued to impact on levels of violence throughout the study period.

The present finding that there was a reduction in inequalities in hospital admissions for violence may not be solely attributable to measures in Wales. The UK Government's Alcohol Strategy 2012,^32^ the Police Reform and Social Responsibility Act 2011,^33^ and the Violence against Women and Girls strategy 2010^34^ may have all contributed in the latter years of the study. Internationally, the WHO operates a global programme to promote a public health approach to violence prevention. For example, the first world report on violence and health^35^ is aimed at researchers and practitioners. The experience of several countries in the region demonstrates that public policy, and sustained approaches that address the underlying causes of violence, can make countries safer.

To the best of our knowledge, this is the first UK study to examine the site of injury resulting in assault-related emergency admissions. A study in India^36^ conducted during a period of civil unrest reported that of patients attending an ED for violence, over one-third were due to head injuries (40%), one-third limb injuries, and around one-eighth chest and abdominal injuries. Our study found a higher proportion of head injuries, particularly among males, perhaps reflective of the timing and nature of the injuries that occurred in the Indian study. Assault has been reported as the most common cause of maxillofacial injuries,^37^ particularly among European countries.^38^ In a Finnish study examining types of maxillofacial injury,^39^ 74% of patients had a fracture, with nasal and orbital fractures the main type. Other studies of maxillofacial injuries have reported contusions and abrasions to be of frequent occurrence,^40^ ^41^ while lacerations were reported more frequently in severe trauma episodes resulting from traffic accidents and gunshot injuries.^42^ In the present study, head injuries were more common among males, a finding supported by studies in India,^37^ Japan,^43^ Iran,^44^ Turkey^45^ and Finland^39^ among other countries.^38^ ^46–48^ Although fractures are common, particularly among males, elsewhere it was been reported that soft tissue injuries were most common in cases of domestic violence.^40^ This research may help explain the finding from the present study that females had a lower proportion of fractures but a higher proportion of superficial and open wounds.

### Policy and practice implications and recommendations

Importantly, the largest decrease in admission rates over the study period was observed in the most deprived quintile. Despite the observed reduction in inequality, violence remains strongly associated with deprivation. There is therefore potential for a greater reduction given that violence is mostly preventable.

This study identifies the types of communities most at-risk who would benefit from interventions, and the time of life at which implementation is required in order to prevent violent assaults. This is a continuing important matter for public health and government. Violence impacts not only the physical health of those assaulted, but can have long-term impacts on mental health and the communities in which victims live.

Historically, the majority of violence responses have used either criminal justice or ED data on assault location to inform operational practice. However, prevention requires a long-term strategic approach. Identifying residential communities with a higher prevalence of individuals who experience violence, and where violent tendencies develop, allows for targeted early prevention. There are programmes that can help support parents, infants and young children during the critical early years such as Nurse Family Partnership^49^ ^50^ and Triple P Positive Parenting Programme.^51^ These programmes, where found to be effective, have been associated with cost-savings.^50–53^ Importantly, such programmes require targeted application in the most deprived areas, and this should be a priority for those involved in violence prevention. Although there are policies in Wales that aim to address violence, including the recent ‘Framework for Managing the Night-Time Economy in Wales’,^54^ the Well-being of Future Generations (Wales) Act,^55^ which provisionally includes a national indicator around ‘feeling safe in the community’, and the Violence Against Women, Domestic Abuse and Sexual Violence (Wales) Act (2015),^56^ more can be done to address key risk factors through other measures, including policy.^1^ Violence is strongly linked to social determinants such as unemployment; income and gender inequality; limited educational opportunities; and cultural, social and gender norms.^1^ Any comprehensive violence prevention strategy must recognise the influence of such factors and identify ways to mitigate or protect against risks. Few countries identified in the international WHO global status report address violence through social and educational policy measures.^1^

We found that the greatest increase in violence was seen between 15 and 24 years of age, particularly in the most deprived communities. Violent responses observed in younger age groups are likely to be a culmination over the preceding years of adaptations and learnt behaviours that manifest during adolescence. In addition to parenting and other interventions, educational and social policy has the potential to mitigate negative experiences in childhood. Critically, in areas with high socioeconomic deprivation, this age group is often accompanied by childbirth and early years parenting. Such violence can contribute to adverse childhood experiences, which have been linked to a wide range of negative health and social outcomes throughout the life course.^9^ The years between 15 and 24 are also the period associated with increasing alcohol consumption.^57^ ^58^ Excessive alcohol consumption is strongly associated with violence, and has been linked with adversity-related hospital admissions for injuries among adolescents.^59^ ^60^ Controlling the availability of alcohol is an effective measure to reduce violence.^61^ The introduction of a minimum unit price advocated in 2013 in ‘an evidence-based alcohol strategy for the UK’,^62^ provides one example of an evidence-based policy measure that could decrease alcohol use and associated violence among youths.^63^ ^64^

Violence prevention and safeguarding policies are widely available, but enforcement is sometimes inadequate. Further action is necessary to strengthen the institutional mechanisms and resources needed to identify violence and ensure appropriate safeguards are in place. Clinician training in screening for child, elder and domestic abuse can help front-line staff to identify violence improve referral pathways, as well as triggering protection services to reduce further incidents.^50^
